# Genomic imbalances are involved in miR-30c and let-7a deregulation in ovarian tumors: implications for *HMGA2* expression

**DOI:** 10.18632/oncotarget.15795

**Published:** 2017-03-01

**Authors:** Antonio Agostini, Marta Brunetti, Ben Davidson, Claes G Tropé, Sverre Heim, Ioannis Panagopoulos, Francesca Micci

**Affiliations:** ^1^ Section for Cancer Cytogenetics, Institute for Cancer Genetics and Informatics, The Norwegian Radium Hospital, Oslo University Hospital, Oslo, Norway; ^2^ Centre for Cancer Biomedicine, University of Oslo, Oslo, Norway; ^3^ Department of Pathology, The Norwegian Radium Hospital, Oslo University Hospital, Oslo, Norway; ^4^ Faculty of Medicine, University of Oslo, Oslo, Norway; ^5^ Department of Gynecology, The Norwegian Radium Hospital, Oslo University Hospital, Oslo, Norway

**Keywords:** HMGA2, miR-30c, let-7a, FHIT, LIN28A

## Abstract

The High-mobility group AT-hook 2 protein (HMGA2) is involved in different processes during tumorigenesis. High expression levels of *HMGA2* are found in various types of cancer, with recent studies highlighting the important role of miRNAs in the regulation of *HMGA2* expression. We report a study of 155 ovarian tumors (30 sex-cord stromal tumors, 22 borderline tumors, and 103 carcinomas) analyzed for *HMGA2* expression as well as the expression of two miRNAs targeting this gene, let-7a and miR-30c. We also evaluated the expression of the fragile histidine triad (*FHIT*) and lin28 homologues (*LIN28A/B*) genes which are known to be an enhancer of miR-30c expression and a repressor of let-7a, respectively. *HMGA2* was found expressed at high levels in most samples analyzed, with clear cell carcinomas as the only exception. let-7a and miR-30c were highly deregulated in all tumor types. *LIN28A* and *FHIT* were found overexpressed in all examined tumor types. The chromosomal imbalances that might lead to loss of the genes expressing let-7a and miR-30c could be evaluated on the basis of previously generated karyotypic and high resolution comparative genomic hybridization (CGH) data on 103 tumors. 76% of the samples with an imbalanced genome had at least one chromosomal aberration leading to a deletion of a miRNA cluster for let-7a and miR-30c. FISH using locus specific probes for these clusters validate the aberrations at the gene level. Our study shows that genomic imbalances are involved in miR-30c and let-7a deregulation. One can reasonably assume that dysregulation of these miRNAs is a cause leading to *HMGA2* upregulation in ovarian tumors.

## INTRODUCTION

Tumors of the ovary are a heterogeneous group which is divided into many different subentities based on histologic and cytologic features. Most ovarian tumors (about 90%) are epithelial in nature. Malignant epithelial ovarian tumors are currently divided into high-grade serous, low-grade serous, endometrioid, mucinous, and clear cell carcinomas [1]. Each carcinoma histotype is characterized by different genetic aberrations. Serous high-grade carcinomas are associated with *TP53* and *BRCA* mutations, whereas their low-grade counterparts often carry *KRAS* and *BRAF* mutations. *KRAS* and *HER2* mutations are frequent in mucinous carcinomas whereas *ARID1A* is frequently mutated in endometrioid and clear cell carcinomas [2]. Complex chromosomal rearrangements, involving chromosome bands 19p13, 19q23, 8q23, and 22q in decreasing order of frequency, are seen in most types of ovarian carcinoma [3]. Borderline tumors of the ovary are neoplasms of low or uncertain malignant potential. They present cellular atypia but are not invasive [4]. Sex-cord stromal tumors account for 8% of all ovarian tumors; thecofibromas and fibromas are among the most common tumors of this type [5] whose genetic features remain largely unknown. Nevertheless, some nonrandom chromosomal aberrations have been reported with trisomy and/or tetrasomy 12 as the most common aneuploidies in thecofibromas, followed by trisomy for chromosomes 10, 18, 4, and 9 [6].

Altered expression of the high-mobility group AT-hook protein gene, *HMGA2*, has been reported in both benign and malignant ovarian tumors [7, 8]. *HMGA2* is usually expressed during embryonic development [9] but not in adult normal tissues [10]. The gene is assumed to be involved in several different tumorigenic processes from cellular proliferation to epithelial-mesenchymal transition and invasive growth [11]. Lately, the role of specific miRNAs has been highlighted in the regulation of *HMGA2* expression in neoplastic cells [12–14]. MicroRNAs are non-coding RNAs with diverse biological functions [15]. They play an important regulatory role by targeting specific mRNAs for degradation or translation repression [16]. This may be the main event in the development of some cancers as many transcripts are affected with profound influence on signaling pathways [17].

To obtain more insight into the role of *HMGA2* and its possible regulation by miRNAs in ovarian malignancies, we analyzed a series of 155 ovarian tumors of different histologies for *HMGA2* gene expression as well as for the expression of two miRNAs targeting *HMGA2*, let-7a and miR-30c. We also evaluated the expression of the fragile histidine triad (*FHIT*) and the lin28 homologues (*LIN28A* and *LIN28B*) genes, which are known to enhance miR-30c expression and repress let-7a, respectively [13, 18].The findings were collated with the pattern of genomic imbalances previously detected in the majority of these tumors (*n* = 103) by means of karyotyping and/or high resolution comparative genomic hybridization (HR-CGH). FISH with locus specific probes was used to validate these results at gene level.

## RESULTS

**Figure 1:**
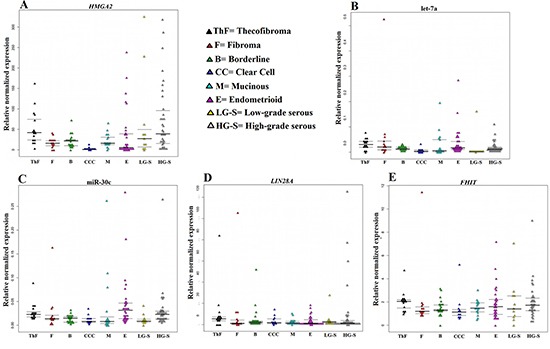
Relative normalized expression of *HMGA2*, *LIN28A*, and *FHIT* as well as the miRNAs miR-30c and let-7a The relative expression of *HMGA2* (**A**)*, LIN28A* (**D**), *FHIT* (**E**) and the miRNAs let-7a (**B**) and miR-30c (**C**) is correlated with histotype.

All molecular investigations gave informative results. The expressions of the genes and miRNAs were normalized using normal, commercially available tissue samples from the ovary. *HMGA2* was expressed at high level in the majority of tumors analyzed (Figure 1). The highest *HMGA2* relative normalized expression levels were found in tumors of the serous subgroup where HG-S had a mean value of 74.3 and a median of 31, whereas the LG-S showed a mean value of 67.6 and a median of 27.2. The serous ovarian carcinomas were followed by the thecofibromas (mean = 53.1; median = 42) and the endometrioid (mean = 36; median = 5) carcinomas. The borderline tumors and the mucinous carcinomas both showed a mean of 21 with a median of 21 and 16, respectively, whereas the fibroma subgroup showed a mean = median = 16. The lowest levels of *HMGA2* expression were detected in clear cell carcinomas (mean = 1.8; median = 0.5).

let-7a and miR-30c were highly deregulated in the ovarian tumors with low levels of expression being found for both miRNAs in all tumor types examined (Figure 1). *LIN28A*, on the other hand, was overexpressed in all histotypes (Figure 1) with the highest expression being seen in HG-S (mean = 10.6; median = 2.32) followed by thecofibromas and fibromas, both with a mean of 10 and a median of 5 and 1.5, respectively. The group of borderline tumors showed a mean value of 5.7 (median = 2.09). The remaining histotypes showed a mean relative expression of 4.3 for LG-S, 3.8 for clear cell, 3.6 for endometrioid, and 2.5 for mucinous carcinomas. *LIN28B* expression was not detected in any of the samples analyzed nor was it seen in the normal controls. *FHIT* was slightly overexpressed in all ovarian tumor types. The highest expression levels for the gene were found in thecofibromas (mean = 2.4; median = 2) followed by HG-S (mean = 2; median = 1.7), LG-S (mean = 2; median = 1.5), endometrioid carcinomas (mean = 1.9; median = 1.6), fibromas (mean = 1.9; median = 1.1), mucinous (mean = 1.5; median = 1.4), borderline (mean = 1.5; median = 1.3), and clear cell carcinomas (mean = 1.4; median = 1.1) (Figure 1).

In order to find out if genomic imbalances could explain the low expression levels of these miRNA, we went back to our previously generated cytogenetic data (karyotype and/or HR-CGH) available on 103 of the 155 samples [3] to see if the region where the let-7a and miR-30c clusters are located were visibly lost. We found a deletion corresponding to at least one of the chromosomal bands where the three clusters for let-7a map-9q22.32, 11q24.1, and 22q13.31-in 47 out of 62 tumors with an abnormal genome (76% of the tumors; Supplementary Table 1).

The most frequent deletion was of chromosomal subband 22q13.31 where *MIRLET7A3* maps (39 out of 47 samples with deletion of the let-7a cluster, 83%). *MIRLET7A1*, located in chromosomal subband 9q22.32, was deleted in 49% of the samples with deletion of the let-7a cluster, while *MIRLET7A2* (11q24.1) was deleted in 19%. Moreover, the bands where the two clusters for miR-30c (*MIR30C1/2)* map, 1p34.2 and 6q13, were found deleted in 41 out of 62 cases with genomic aberrations (66%). *MIR30C1* on 1p34.2 had a rate of loss of 88% (36 out of 41), while *MIR30C2* (6q13) was deleted in 32% (13 out 41) of the samples with a miR-30c cluster deletion.

**Figure 2:**
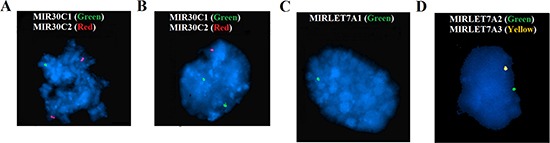
FISH analyses of the let-7a and miR-30c clusters A signal pattern indicating heterozygous deletion is seen of *MIR30C1* on 1p34.2 (**A**), *MIR30C2* on 6q13 (**B**), *MIRLETA1* on 9q22 (**C**), *MIRLET7A2* on 11q24, and *MIRLET7A* on 22q13 (**D**).

We performed FISH analyses using locus specific probes for the let-7a and miR-30c clusters on 42 cases, 22 of which had not been cytogenetically characterized, to confirm at the genic resolution level that loss of one or more clusters had indeed occurred. FISH experiments gave informative results in all but four tumors (38 out of 42). Twelve cases showed a normal diploid pattern of signals for the all probes analyzed. The remaining 26 cases showed mostly heterozygous deletions of one or more miRNAs (Figure 2); only in four cases did the signal pattern indicate a homozygous deletion. At least one let-7a cluster was deleted in 88.5% (23 out of 26) tumors with abnormalities. *MIRLET7A3* on 22q13.31 was deleted in 91% of the cases with a deleted let-7a cluster (21/23), while the deletion rate for *MIRLET7A2* (11q24.1) and *MIRLETA1* (9q22) was 21% (5/23) and 30% (7/23), respectively. At least one mir-30c cluster was deleted in 19% (5/26) of the cases analyzed. *MIR30C1* on 1p34.2 was deleted in 2 out of 5 cases with a miR-30c cluster deletion, while *MIR30C2* on 6q13 was deleted in 4 out of 5 cases.

## DISCUSSION

The tumorigenic mechanisms behind different ovarian malignancies are still poorly understood, as is tumor progression leading to recurrences and/or metastases. In a previous study [7], we found *HMGA2* expressed in 74% of the ovarian tumors examined. We have now quantified the expression of this gene in the same series of tumors.

HMGA2 belongs to the High-mobility group AT-hook family of non-histonic proteins involved in a wide variety of nuclear processes from chromatin dynamics to gene regulation. The gene is expressed during embryonic development [9] but is usually silent in adult normal tissues except adipose tissues, lung, and kidney [10]. High expression levels of *HMGA2* have been found in various tumors, both benign and malignant. In the present series, the gene was found highly expressed in all tumor types analyzed; however, the level differed among the different histological subgroups. Serous ovarian carcinomas showed the highest expression, with HG-S generally demonstrating higher levels than did the LG-S subgroup. Although this certainly indicates a general correlation between expression level and how malignant the tumor is, exceptions were seen. Whereas *HMGA2* was expressed at high levels in the sex-cord stromal tumors analyzed (fibromas and thecofibromas), tumors that have hitherto not been subjected to this type of examination, clear cell carcinomas were found to show the lowest expression level despite these tumors’ obvious malignant potential.

The mechanisms that lead to expression of *HMGA2* are still not fully understood, but interaction between miRNAs and the *HMGA2* 3′untranslated region (3′UTR) seems to play a crucial role. It has been shown that the *HMGA2* 3′UTR has many regulatory sequences which are targeted by different families of miRNAs [19], and it is thought that miRNA-dependent repression is the main mechanism for controlling *HMGA2* expression [12–14]. Another piece of evidence for the importance of the interaction between HMGA2 and miRNAs is the many disrupted forms of *HMGA2*, due to rearrangements of chromosomal band 12q15, that are seen in different benign mesenchymal tumors [20–22]. These alterations involve exon 3 and cause deletion of downstream regions leading to a truncated transcript that can evade miRNA-dependent gene silencing. Since we have previously shown that truncation of *HMGA2* is not common in ovarian malignant tumors [7], we in this study focused on other causes of *HMGA2* deregulation analyzing two miRNAs that are known to target *HMGA2* and suppress its function, miR-30c and let-7a. Deregulation of the former has been identified as the main mechanism of *HMGA2* regulation in lung cancer [13] whereas similar involvement of the latter was found in other tumors such as nasopharyngeal carcinoma and non small cell lung cancer [23, 24]. Both miRNAs were found low expressed in all the ovarian tumors analyzed with no major group-to-group differences. Each of these miRNAs is regulated in a different manner: miR-30c expression is enhanced by the Fragile Histidine Triad protein (FHIT), whereas let-7a is repressed by LIN28A/B. Since it is not known which of these two pathways is active in ovarian cancer, we investigated both and found *FHIT* gene expressed in all tumors. This is a well known tumor suppressor gene [25] whose expression has been shown to exhibit an inverse correlation with *HMGA2* in lung cancer [13], where *FHIT* enhances the expression of miR-30c and causes *HMGA2* repression. This does not seem to be the case for ovarian tumors as both *FHIT* and *HMGA2* were found expressed in all tumors analyzed. Another path known to regulate *HMGA2* expression involves the miRNAs belonging to the let-7 family that are frequently downregulated in cancer, usually resulting in *HMGA2* overexpression [26, 27]. In all tumors analyzed, we found low expression of let-7a and corresponding overexpression of *LIN28A*. Allegedly, the downregulation of the let-7 family of miRNAs is caused by overexpression of the RNA binding proteins homologues LIN28A and/or LIN28B that inhibit the maturation of both pri- and pre-let-7 [28]. This seems to be the case also for ovarian tumors where the pathway leading to *HMGA2* deregulation is relying on overexpression of LIN28A, downregulating let-7a and leading to overexpression of *HMGA2*. We found that *LIN28A* showed an average normalized expression of 2 in almost all groups, while let-7a showed a normalized expression close to zero.

CGH data showed deletions involving the clusters for let-7a and miR-30c in 76% and 66% of the tumors with a rearranged genome, respectively. FISH analyses validated at the genic resolution level the above data and showed that the deletions were heterozygous in the majority of samples. This fits well with what was also reported by Kan et al. 2015 [29] who found that genomic alterations led to dysregulation of miRNA expression in serous ovarian carcinomas. We now show that this also holds for other types of ovarian cancer. Deletions of let-7a clusters were found in all four fibromas analyzed with FISH. Taken together, the CGH and FISH data show that, in borderline tumors, genomic deletion of at least one of the clusters was seen in six out of seven cases, while the same pattern was seen in two out of three clear cell carcinomas, in five out of eight mucinous carcinomas, in 10 out of 17 endometrioid carcinomas, in all three LG-S, and in 28 out of 33 HG-S. Interestingly, *MIRLET7A3* on 22q13.31 was found frequently deleted in ovarian tumors, in 62% of the cases analyzed by CGH (39/62) but 81% of the cases analyzed by FISH (21/26). Our results indicated that this cluster is deleted across the entire spectrum of ovarian tumors: from benign sex-cord stromal tumors to carcinomas, underscoring yet again the importance of dysregulated miRNAs in ovarian tumorigenesis and progression. Further analysis of larger cohorts of ovarian tumors of different types/histologies should clarify the extent to which genomic losses act through altering miRNA function.

In conclusion, our study shows that the miRNAs let-7a and miR-30c are deregulated in ovarian cancer, and that genomic imbalances may be a cause of this deregulation. Although we suppose that the low levels of let-7a in ovarian tumors are brought about by deletions of let-7a clusters, we cannot exclude that also *LIN28A* may play a role in the downregulation of this miRNA when expressed (Figure 1). *FHIT* does not seem to enhance miR-30c expression in ovarian tumors, so probably other causes act together with genomic imbalances leading to miR-30c deregulation. The high frequency of downregulation of these miRNAs found indicates that this is a significant way of obtaining *HMGA2* deregulation in ovarian tumors. Our data nevertheless lead us to suppose that also other players are involved in *HMGA2* regulation, not least because although the let-7a and miR-30c levels differ but little among the various tumor histotypes, the levels of *HMGA2* expression do. The fact that *HMGA2* is not frequently expressed in clear cell carcinomas although this is one of the tumor groups with the lowest expression of the miRNAs, suggests some other type of *HMGA2* regulation in at least this ovarian cancer subgroup.

## MATERIALS AND METHODS

### Tumor material

The material consisted of fresh frozen samples from 155 ovarian tumors surgically removed at The Norwegian Radium Hospital between 1999 and 2010. The series consists of 30 sex-cord stromal tumors (16 thecofibromas, ThF; 14 fibromas, F), 22 borderline tumors (B), and 103 carcinomas of which 12 were of the clear cell (CC) histological subtype, 16 were mucinous (M), 30 endometrioid (E), 10 low-grade (LG-S) serous, and 35 high-grade (HG-S) serous carcinomas. The study was approved by the regional ethics committee (Regional komité for medisinsk forskning-setikk Sør-Øst, Norge, http://helseforskning.etikkom.no) and written informed consent was obtained from the patients.

### RNA extraction

Total RNA was extracted using miRNeasy Kit (Qiagen, Hilden, Germany) and QIAcube (Qiagen). The concentration and purity of the RNA was measured with a Nanovue Spectrophotometer (GE Healthcare, Pittsburgh, PA, USA).

### Real-time polymerase chain reaction (real-time PCR)

The expression of the miRNAs and genes of interest was assessed by Real-Time PCR using the CFX96 Touch Real-Time PCR detection system (Bio-Rad Laboratories, Oslo, Norway). The reactions were carried out in quadruplicate using TaqMan Assays and the TaqMan Universal Master Mix II with UNG (Applied Biosystems, Foster City, CA, USA) following the manufacturer's protocol. Human Universal Reference Total RNA (Clontech, Mountain View, CA, USA) was used as internal reaction control. Two commercial Total RNAs from the ovary, MVP Total RNA Human Ovary (Agilent Technologies, Santa Clara, CA, USA) and Human Ovary Total RNA (Zyagen, San Diego, CA, USA), were used as reference for relative expression normalization. The Real-Time data were analyzed with Bio-Rad CFX manager 3.1 (Bio-Rad). The normalized expression was calculated using the 2^−ΔΔCt^ (Livak) method [30].

### microRNA expression

Ten ng of total RNA were reverse transcribed with the TaqMan MicroRNA Reverse Transcription Kit (Applied Biosystems) following the manufacturer's protocol. miRNA expressions were assessed with Real-Time PCR using the TaqMan MicroRNA Assays (Applied Biosystems) for let-7a (TM:000377) and miR-30c (TM:000419), whereas RNU6B (TM:001093) was used as reference.

### Gene expression

One μg of extracted total RNA for each tumor was reverse-transcribed in a 20 μl reaction volume using iScript Advanced cDNA Synthesis Kit according to the manifacturer›s instructions (Bio-Rad Laboratories, Oslo, Norway). Gene expression was assessed with Real-Time PCR using the TaqMan Gene Expression Assays (Applied Biosystems) for *HMGA2* (Hs_04397751_m1), *FHIT* (Hs_00179987_m1), *LIN28A* (Hs_00702808_s1), and *LIN28B* (Hs_01013729_m1), whereas *RPL4* (Hs_01939407_gH) was used as a reference gene as it has been proven to be stably expressed in ovarian cells [31, 32].

### Fluorescence *in situ* hybridization (FISH)

FISH analyses were performed on interphase nuclei. Bacterial Artificial Chromosomes (BAC) clones were retrieved from the RPCI-11 Human BAC and CalTech Human BAC libraries (P. De Jong Libraries;http://bacpac.chori.org/). The BAC clones were selected according to physical and genetic mapping data as reported on the Human Genome Browser at the University of California, Santa Cruz website (May 2004,http://genome.ucsc.edu/). The clones used were: RP11-2B6 for MIRLET7A1 (9q22.32), RP11-453C14 for MIRLET7A2 (11q24.1), CTD-2504J8 for MIRLET7A3 (22q13.31), RP11-170L4 for MIR30C1 (1p34.2), and RP11-756H9 for MIR30C2 (6q13). All clones were grown in selective media, and DNA was extracted and labelled according to the manufacturer's recommendations (http://bacpac.chori.org/). The slides were counter-stained with 0.2 *μ*g/ml DAPI and overlaid with a 24 × 50 mm^2^ coverslip. Fluorescent signals were captured and analyzed using the CytoVision system (Leica Biosystems, Newcastle, UK).

## SUPPLEMENTARY MATERIALS FIGURES AND TABLES




