# Supplementary material for: Genomic imbalances are involved in miR-30c and let-7a deregulation in ovarian tumors: implications for HMGA2 expression
Source: Oncotarget. 2017 Mar 1;8(13):21554–60. doi: 10.18632/oncotarget.15795 (PMC5400605; doi:10.18632/oncotarget.15795)
Supplement: Supplementary file 1 [file oncotarget-08-21554-s001.pdf]

Genomic imbalances are involved in miR-30c and let-7a deregulation in ovarian tumors: implications for *HMGA2* expression

Supplementary Materials

Supplementary Table 1: Overview of samples with genomic imbalances involving miR-30c and let-7a clusters. See Supplementary_Table_1
