# Supplementary material for: Genomic imbalances are involved in miR-30c and let-7a deregulation in ovarian tumors: implications for HMGA2 expression
Source: Oncotarget. 2017 Mar 1;8(13):21554–60. doi: 10.18632/oncotarget.15795 (PMC5400605; doi:10.18632/oncotarget.15795)
Supplement: Supplementary file 2 [file oncotarget-08-21554-s002.docx]

**Table 1 Overview of samples with genomic imbalances involving miR-30c and let-7a clusters.**

|  | **Case number** | **Diagnosis** | **Karyotype** | **Genomic imbalances (CGH)** |
| --- | --- | --- | --- | --- |
| 1 | 08-1485 | Thecofibroma | 46,XX[83] | NA |
| 2 | 08-1649 | Thecofibroma | 46,XX[53] | NA |
| 3 | 09-200 | Thecofibroma | 45,XX,del(16)(q23),-22[13]/46,XX[2] | NA |
| 4 | 07-1013 | Fibroma | 46,XX[72] | NA |
| 5 | 01-642 | Borderline | 47,XX,+12[4]/47,XX,+7[3]/45,XX,-6[3]/ 46,XX[63] | rev ish enh(**6q12q21**),dim(**1p31pter**,**11q23**, **22q11q13**) |
| 6 | 02-01 | Borderline | 46,XX[16]/92,XXXX[23] | rev ish dim(**1p32pter**,**9q22**,**22q11q13**) |
| 7 | 02-828 | Borderline | 46,XX[13]/92,XXXX[7] | rev ish dim(**1p32p36**,**22q11q13**) |
| 8 | 03-325 | Borderline | 46,XX[15] | rev ish dim(**1p31pter**,**22q11q13**) |
| 9 | 04-831 | Borderline | 46,XX[84] | rev ish dim(**22q**) |
| 10 | 04-832 | Borderline | 47,XX,+7[18] | rev ish enh(**6q12q21**),dim(**1p32pter**,**22q**) |
| 11 | 04-1213 | Borderline | 46,XX[3] | rev ish dim(**1p34p35**) |
| 12 | 00-20 | Clear Cell | 75,der(1)del(1)(p34)del(1)(q32),inc[2]/131~146,idemx2[2]/ 46,XX[12] | rev ish enh(**1p34p35**) |
| 13 | 03-268 | Clear Cell | 63~85<3n>,del(X)(q22),add(X)(q28), +1,i(1)(q10)x2, add(1)(p34),-2,del(3) (q21),-4,add(5)(p11),-6,add(6)(p25), +7,i(8)(q10),-10,add(11)(q22),i(11)(q10),add(19)(p13)x2,-21, inc[8]/46,XX[2] | rev ish dim(**1p32p36**,**6q13q15**, **9q22**,**11q23**, **22q12q13**), |
| 14 | 08-965 | Clear Cell | 66~70,XX,der(1)t(1;7)(p21;p15),+del(1)(p11),?del(3)(q11), ?del(6)(q23),add(7)(p22),add(8)(q24),?del(9)(q11),add(11) (p15),dic(12;19)(p11;q13),add(16)(q24),add(19)(p13),add(19) (q13),inc[cp7]/46,XX[2] | NA |
| 15 | 00-780 | Mucinous | Culture failure | rev ish dim (**1p32p35**,**22q11q13**) |
| 16 | 01-582 | Mucinous | 84~90,XXXX,add(7)(q32)x2,add(13)(p13)x2,add(15)(p13), -16, i(17)(q10),add(22)(p13)[15] | rev ish enh(**6q13q16**), dim(**1p32pter**,**9q22q31**,**22q**) |
| 17 | 01-700 | Mucinous | 46,XX[116] | rev ish dim(**1p34p35**,**22q11q12**) |
| 18 | 07-744 | Mucinous | 46,XX[65] | NA |
| 19 | 08-1650 | Mucinous | 46,XX,del(1)(q21)[2]/46,XX[88] | NA |
| 20 | 00-752 | Endometrioid | 49~51,XX,del(6)(q15q23),+3~5mar[3]/ 88~95,idemx2[5] | rev ish dim(**1p33p34**) |
| 21 | 00-1168 | Endometrioid | Culture failure | rev ish dim(**9q21qter,22q11q13**), |
| 22 | 01-196 | Endometrioid | 46,XX[16] | rev ish dim**(1p32p36,9q22,22q11q13)** |
| 23 | 01-987 | Endometrioid | Culture failure | rev ish dim(**1p32pter**,**9q22**,**11q22q25**,**22q11q13**) |
| 24 | 02-50 | Endometrioid | 37~38,inc[2]/46,XX[91] | rev ish enh**(6q12q21),** dim**(1p33p36,9q,22q11q13)** |
| 25 | 04-1141 | Endometrioid | 46,XX[4] | rev ish dim(**1p13pter**,**6q12qter**,**22q**) |
| 26 | 08-95 | Endometrioid | 46,XX | NA |
| 27 | 08-746 | Endometrioid | 51,XX,+i(1)(q10),+2,+7,+10,+20[15] | NA |
| 28 | 08-753 | Endometrioid | 71~74,XXX,+5,+7,+8,+9,+10,-11,+12, -13,+16,-18,-19, +20[19] | NA |
| 29 | 08-1679 | Endometrioid | 55~64,XXX,add(1)(p11),add(1)(p36),add(1)(p21), +del(1)(p21),add(2)(p11),add(3)(p11),del(6)(q21),+7,add(19) (p13),der(19)t(6;19)(p11;p13)?+20,+mar,inc[cp15] | NA |
| 30 | 09-167 | Endometrioid | NA | NA |
| 31 | 00-626 | LG Serous | Culture failure | rev ish enh(**6q15q16**), dim(**1p31pter**,**9q22qter,22q11q13**) |
| 32 | 04-1261 | LG Serous | Culture failure | rev ish dim(**1p13pter,6q12qter**,**9q21qte**r, **22q11q12**) |
| 33 | 55-99 | HG Serous | 72~90,XXX,del(1)(q11),der(1)t(1;5)(q43;q11),der(1;16) (q10;p10),i(5)(p10),add(6)(q13),del(6)(q15),del(11)(q12), der(11)t(11;17)(p15;q12),add(12)(p13),i(13)(q10),inc[cp24]/ 46,XX[4] | rev ish enh(**1p35pter**),dim(**9q21q22**,**22q13**) |
| 34 | 60-99 | HG Serous | 54~61,add(1)(p21),del(1)(q21),add(4)(p11),add(6)(q27)x2, add(7)(p22)x2,del(12)(p11)x2,+13,der(13;14)(q10;q10), add(15)(p11),add(19)(p11),inc[cp13]/46,XX[3] | rev ish enh**(1p36)** |
| 35 | 00-39 | HG Serous | 35~42,del(1)(q21),add(1)(p13),add(6) (q22),inc[cp9]/71~87,idem x2[cp10]/ 46,XX[3] | rev ish enh(**1p13p35**),dim(**9q13q34**) |
| 36 | 00-55 | HG Serous | 39,X,-X,i(3)(q10),-4,add(5)(q13), i(5)(p10),-6,add(7)(p13), add(10)(p11),add(11)(p11),der(13;14)(q10;q10),-17,-21[13]/ 78,idemx2[3]/46,XX[3] | rev ish enh(**1**),dim(**22q**) |
| 37 | 00-62 | HG Serous | culture failure | rev ish enh(**6q13qter**), dim(**1p34p36,9q21qter,22q12q13**) |
| 38 | 00-94 | HG Serous | 46,XX[6] | rev ish enh(**6q13q21**),dim(**9q22,22q**) |
| 39 | 00-129 | HG Serous | 87~141,dup(1)(q21q31),add(3)(q25),add(4)(q31), add(6)(q21),inc[3]/46,XX [13] | rev ish dim(**1p35p36**,**6q**,**22q11q13**) |
| 40 | 00-206 | HG Serous | 116~162,add(4)(p15),add(5)(p13),del(7)(q31),der(8)t(8;13) (p23;q14),i(9)(q10),add(11)(p15)x2,add(15)(p11)x2, inc[6]/46,XX[94] | rev ish dim(**22q**) |
| 41 | 00-522 | HG Serous | 46~49,add(11)(q22),add(14)(p11),add(16)(q23),add(19)(q13),inc[6]/89~94,idemx2[2] | rev ish enh-- dim-- |
| 42 | 00-672 | HG Serous | 59~62,inc[5] | rev ish dim(**1p33pter**,**9q**,**22q12qter**) |
| 43 | 00-801 | HG Serous | 63~106,add(4)(q34),add(6)(q12),add(14)(p11),inc[3]/ 46,XX[10] | rev ish enh(**6q12q21**),dim(**22q**) |
| 44 | 01-163 | HG Serous | 46,XX[4] | rev ish enh(**6q12q21**),dim(**1p34pter**,**22q12q13**) |
| 45 | 01-169 | HG Serous | 46,XX[48] | rev ish enh(**6q15q16),** dim(**1p32p36,9q22,22q11q13**) |
| 46 | 01-295 | HG Serous | 42~45,XX,t(2;11)(q31;q14),+7,+14,-20,add(20)(q13), +mar[7]/83~85,XXXX, idemx2[4]/46,XX[1] | rev ish enh(**6q15q16**),dim(**1p32p36**) |
| 47 | 01-548 | HG Serous | 55~80,inc[8]/46,XX[11] | rev ish dim(**6q16q27**,**9q21qter**,**22q**) |
| 48 | 01-1000 | HG Serous | Culture failure | rev ish enh(**1p35p36**,**6q16q23**),dim(**11q23q25**) |
| 49 | 01-1020 | HG Serous | 46,XX[3] | rev ish enh(**1p31pter**), dim(**6q12q14**,**11q23q25**,**22q**), |
| 50 | 02-79 | HG Serous | 46,XX[3] | rev ish enh(**6q12q24**),dim(**1p33p36**,**22q12q13**) |
| 51 | 02-93 | HG Serous | 46,XX[93] | rev ish enh(**6q12q22**), dim(**1p32pter**,**9q**,**22q11q13**) |
| 52 | 02-233 | HG Serous | 62~71,XX,del(1)(p32),add(5)(p13),add(12)(p11),add(19) (q13),inc[cp8]/46,XX[2] | rev ish enh(**6q12q21**),dim(**9q22q31**) |
| 53 | 02-248 | HG Serous | 59~63,XXX,add(2)(q36),add(3)(q28),add(5)(p15),add(19) (p13),der(19)add(19)(p13)add(19)(q13),inc[2]/46,XX[5] | rev ish dim(**22q**) |
| 54 | 02-333 | HG Serous | 47~49,XX,+8,+9[2]/49,idem,+5,-6,+7[4]/54,idem,+3,+5,+6, +7,+14,+17, +19[5] | rev ish enh(**1p36**),dim(**6**) |
| 55 | 02-363 | HG Serous | 65~68,XX,-X,+1,+2,del(3)(p13),+4,add(4)(p12)x2,add(5) (p15),-6,+7,add(7)(p15),-8,-9,-12,-13,-14,-15,-17,-18, der(19)add(19)(p13)add(19)(q13),+20,+20,-21,-22,+2mar[9] | rev ish dim(**6**,**9q21qter**,**22q**) |
| 56 | 02-466 | HG Serous | 73~81,del(1)(p22),add(1)(q12),add(4)(p11),del(6)(q15), i(8)(q10),add(11)(q22),inc[6] | rev ish dim(**1p34pter**,**9q**,**22q**) |
| 57 | 03-420 | HG Serous | 34~40,del(1)(p32)x2,-2,-2,-3,del(3) (p21),-5,-5,del(6)(q23), +del(7)(q31), add(7)(p21), -8,-8,-9,-9,-10,add(11)(q22), inc[27]/46,XX[8] | NA |
| 58 | 04-86 | HG Serous | 52~54,del(1)(q12),del(3)(p13),inc[9]/ 46,XX[26] | rev ish enh(**6q12q24**), dim(**9q22qter**,**11q23qter**,**22q12qter**) |
| 59 | 04-464 | HG Serous | 61~73,XXX,+1,der(1)(1qter→1p34:: 1p11→1qter), add(2)(p11),del(2)(q32), der(3)add(3)(p21)add(3)(q29),-4,+5, i(6)(p10)x2,+7,add(7)(q36)x2,-8,del(11)(q22)x3,add(12) (q24),-14,-15,-16,add(17)(p11),-18,-19,-19,-20,-21, +5~15mar[22] | rev ish dim(**1p33pter**,**9q22q31**,**11q23q25**, **22q11q13**) |
| 60 | 04-499 | HG Serous | 46,XX[5] | rev ish enh(**6q12q21**),dim(**1p22pter**,**22q**) |
| 61 | 04-760 | HG Serous | 46,XX[8] | rev ish dim(**9q22qter,22q11q13**) |
| 62 | 04-1019 | HG Serous | culture failure | rev ish dim(**9q22**) |
